# Root Traits and Phenotyping Strategies for Plant Improvement

**DOI:** 10.3390/plants4020334

**Published:** 2015-06-15

**Authors:** Ana Paez-Garcia, Christy M. Motes, Wolf-Rüdiger Scheible, Rujin Chen, Elison B. Blancaflor, Maria J. Monteros

**Affiliations:** The Samuel Roberts Noble Foundation, 2510 Sam Noble Parkway, Ardmore, OK 73401, USA; E-Mails: apaezgarcia@noble.org (A.P.-G.); cmmotes@noble.org (C.M.M.); wrscheible@noble.org (W.-R.S.); rchen@noble.org (R.C.); eblancaflor@noble.org (E.B.B.)

**Keywords:** root, phenotyping, breeding, root breeding, root ideotype, root plasticity, abiotic stress

## Abstract

Roots are crucial for nutrient and water acquisition and can be targeted to enhance plant productivity under a broad range of growing conditions. A current challenge for plant breeding is the limited ability to phenotype and select for desirable root characteristics due to their underground location. Plant breeding efforts aimed at modifying root traits can result in novel, more stress-tolerant crops and increased yield by enhancing the capacity of the plant for soil exploration and, thus, water and nutrient acquisition. Available approaches for root phenotyping in laboratory, greenhouse and field encompass simple agar plates to labor-intensive root digging (*i.e.*, shovelomics) and soil boring methods, the construction of underground root observation stations and sophisticated computer-assisted root imaging. Here, we summarize root architectural traits relevant to crop productivity, survey root phenotyping strategies and describe their advantages, limitations and practical value for crop and forage breeding programs.

## 1. Introduction

Roots provide essential functions including the uptake of water and nutrients for plant growth, serve a role as storage organs, anchor the plants to the soil and are the site of interactions with pathogenic and beneficial organisms in the rhizosphere. The plasticity of root growth and development in response to changing moisture and nutrient status of the soil provides opportunities for exploring natural variation to identify beneficial root traits to enhance plant productivity in agricultural systems [1,2,3]. The spatial distribution of all root parts in a particular growth environment is collectively referred to as root system architecture (RSA). RSA is dynamic and affected by the external environment (soil moisture, temperature, nutrients and pH) and the enclosing microbial communities that impact the way in which a plant detects and responds to its surroundings [4,5]. Different root characteristics enable plants to respond, adapt and thrive in different environments.

An increasing global population requires agricultural production systems and cultivars that can continue to be productive in erratic weather patterns and are capable of more efficient resource capture from the soil. Breeding programs have traditionally focused on the aboveground plant parts (forage, seed or grain production) for the generation of food, feed and fiber. Breeders aim to develop improved cultivars that can tolerate a variety of abiotic stress conditions such as drought or flooding. These approaches include selection of individuals with improved plant growth characteristics such as grain or biomass yield, seed production, leaf surface area, the number of tillers and disease resistance. Strategies to implement “root breeding” require the identification of the underground root traits that enable a plant to more efficiently utilize water and nutrients in different environments. Multiple studies have identified links between root traits and crop productivity [6,7] including performance under drought [8] and grain yield [9]. Understanding the root phenes that result in higher yields and increased stress tolerance would provide tangible targets for breeders to select individuals with the ideal root phenotypes to use as parents and develop breeding lines to advance through the crop improvement process. The success of breeding programs aimed at modifying RSA is dependent on the specific trait under selection in different crops, the heritability of the trait, the ability to accurately and efficiently phenotype roots of multiple genotypes, the specific farming system used (row crops *vs.* swards or pastures), the soil properties and the target set of breeding environments [10].

The key roles of roots as part of plant development have sparked renewed interest in understanding the molecular mechanisms that control RSA in crops [10,11,12,13,14]. Strategies to understand root growth and RSA include forward and reverse genetics using mutants of the model plant species *Arabidopsis thaliana*, *Medicago truncatula* or *Brachypodium distachyon* and the identification of quantitative trait loci (QTL) underlying the natural phenotypic variation in root traits among populations [15,16,17]. Integrating knowledge from the genetic mechanisms that underlie RSA to the identification of ideal root phenotypes for crops growing in a specific environment can enable breeders to select target root traits for existing or reclaimed soils for improved agricultural production.

The root system also contributes to soil health, which refers to the continued capacity of the soil to function as a living ecosystem that sustains plants, animals and ultimately humans [18,19]. Strategies such as crop rotation are beneficial because they disrupt pest and disease cycles, affect nutrient availability and improve soil health [20]. For example, the interaction between root systems of legumes and nitrogen-fixing soil bacteria provide plant-available nitrogen (N) for a subsequent crop grown in a crop rotation system [21,22]. The result is a reduction in the use of chemical fertilizers via the implementation of more environmentally-friendly and sustainable farming practices. Plant roots also keep the soil in place, reduce water leaching and soil erosion and are key for soil phytoremediation. The latter takes advantage of the ability of some plants to extract heavy metals or other toxic compounds with their roots from contaminated soils, and to accumulate and store these in aboveground organs that can be easily harvested and disposed of [23].

Breeding efforts that select for and modify specific root traits are limited despite the importance of the root systems for enhancing the acquisition of water and nutrients [24]. Genetic gains in forage and grain production are important targets for plant breeding programs and these gains could be enhanced by understanding the root traits that contribute to improved plant performance. The challenge is to develop systems for non-destructive root phenotyping to accurately reflect and capture the RSA. These methods should allow continuous monitoring of root development and its response to different growing conditions as well as relatively high-throughput systems to efficiently evaluate a large number of genotypes as part of the breeding program. The goal of this review is to provide an overview of root phenotyping strategies that are used in the laboratory, greenhouse or the field, and range from simple approaches requiring minimal infrastructure to those that involve the use of advanced technologies, all of which can serve as a resource for plant breeders, geneticists and other researchers interested in understanding the genetic factors affecting RSA traits of value for practical crop improvement.

## 2. Key Elements of Root System Architecture Relevant for Crop Productivity

The distribution of nutrients across different layers of the soil and the availability of these nutrients in diferent environments make the RSA a fundamental trait for resource acquisition [25,26,27]. In soils with limited nutrient availability, intensive fertilization is often used to maximize biomass and yield. This approach is expensive for the grower, may result in nutrient leaching into ecosystems and ultimately contributes to the eutrophication of rivers, lakes and coastal waters. More efficient fertilizer uptake is needed to reduce the negative impacts of fertilization, and this can be achieved by using genotypes with roots that are more efficient at capturing nutrients from the soil (e.g., shallow roots for topsoil foraging of phosphorus (P)). Nutrient and water deficiencies can also change the ratio of growth between roots and the aboveground plant organs. Changes in the development and architecture of root systems in response to stress conditions, such as water and nutrient limitation, can be used to identify links between the molecular mechanisms involved in sensing and transduction of the plant signals mediating these changes [26,28,29] (Table 1).

Roots sense and respond to abiotic and biotic stresses, and are able to communicate with the aboveground plant parts via signaling pathways. Root morphology and physiology impact the growth and development of aboveground plant organs through altered root to shoot transport of mineral nutrients or diverse organic signaling molecules including hormones, proteins and RNAs [30]. Low water availability (drought) represents an important abiotic stress resulting in significant crop losses [31] and, therefore, plant roots utilize morphological plasticity to adapt to and respond to soil moisture levels [32,33]. Research areas of practical value include identifying roots traits that increase the capacity of soil foraging for water and maintain productivity during limited water availability. Because specific root traits targeted for plant improvement under drought and nutrient limitation conditions have been the subject of several excellent review articles [34,35,36,37,38,39], we only briefly describe the root traits that are of practical value for crop and forage production systems.

**Table 1:** Relationships between root architecture phenes and environmental factors.

Root Traits/Phenes	Description	References
**Rooting depth**		
Primary root length	Primary root growth is inhibited during P-limitation.	[27]
	A moderately high rate of nitrate supplies can be inhibitory under some culture conditions.	[25]
	Deeper roots provide plants with better access to stored water in the deeper layers of the soil substratum.	[34]
Root tip diameter	Root tips with large diameters have improved root penetration of hard, drying soils.	[40]
Gravitropism	Steeper root angles and more robust seedling gravitropic responses (which translates to deeper root systems) results in plants that are more tolerant to drought.	[8]
**Root hairs**		
Root hairs	Proliferation of root hairs is stimulated in P-limited conditions, root hairs can contribute 70% or more of the total root surface area and can be responsible for up to 90% of P acquired.	[40,41,42]
	Root hairs protect the water status of young root tissue.	[43]
	Root hairs improve root penetration of hard, drying soil.	[40]
Rhizosheaths	These protect the water status of young root tissues.	[44]
	Rhizosheaths can increase zinc uptake from dry soils.	[45]
	Their presence is correlated with the aluminum tolerance of root hairs.	[44]
**Root Branching**		
Length and number of lateral roots (LRs)	LR initiation and emergence is stimulated during P limitation.	[46,47]
	External nitrate stimulates LR initiation and elongation, whereas a high plant internal nitrate/N status inhibits LR growth. Early LR development can also be systemically inhibited by N deficiency. Reduced frequency of LR branching and longer LR improve N capture from low-N soils in maize.	[25,48,49]
	Lateral roots are considered the most active portion of the root system for water uptake and represent the majority of the length and surface area of root systems in various types of plants.	[50]
Shallow/adventitious roots	A reduced gravitropic trajectory of basal roots, adventitious rooting and altered dispersion of lateral roots enable topsoil foraging in response to low P availability. Recombinant inbred lines of common bean with shallow basal roots have better P acquisition in the field. Maize plants with brace and crown roots growing at a more shallow angle are more efficient in N use.	[41,46,51,52,53,54]
Cluster roots	Bottlebrush-like clusters of ephemeral rootlets arising from a persistent mother root. These are characteristic of the *Proteaceae* and several other plant species adapted to extremely low soil fertility. Cluster roots are better able to access nutrients such as P by producing large amounts of exudates containing phosphatases and carboxylates that help release bound P.	[55,56]
Crown root number	Reduced crown root number is associated with greater rooting depth, N capture and yield in low N soil.	[57,58]
**Anatomical root traits**		
Root cortical aerenchyma	Root cortical aerenchyma are induced by drought and N, P or potassium limitation in maize. Their formation reduces respiration, nutrient content of root tissues and the metabolic cost of soil exploration. Root cortical aerenchyma increased rooting depth by 15%–30% that led to a corresponding elevation in N capture and biomass/grain yield under N stress.	[36,54,57,58,59,60]
Cortical cell file number and cell size	Reduced root cortical cell file number and large root cortical cell size improve maize drought tolerance.	[61,62]
Cell wall modification	Suberization/lignification affect radial water conductance, and may be important in reducing water loss from mature roots into dry soil.	[36]
**Metabolic traits**		
Respiration	Reduced root respiration in P-efficient common bean reduces the metabolic cost of soil exploration during P-limitation.	[63,64]

### 2.1. Rooting Depth

Rooting depth is one of the most frequently evaluated traits because crops with deeper roots have better access to stored water and nutrients such as N, a soluble nutrient that tends to leach into the deeper layers of the soil [34] (Table 1). Although rooting depth is strongly influenced by the soil’s physical and chemical properties [39], recent studies have uncovered additional factors that impact rooting depth, which could be exploited for crop breeding programs. Most notable are the anatomical traits in which a strong correlation between a specific type of root anatomy and plant performance under stress has been demonstrated [36,57] (Table 1). For example, maize genotypes with reduced cortical cell file number and large cortical cell size have deeper roots under water stress conditions because of the reduced metabolic costs associated with soil exploration [61,62]. A similar trend was observed in regard to root cortical aerenchyma, the enlarged air spaces in the root cortex, which has been traditionally associated with responses to hypoxia. Similar to the cortical cell file number, the larger root cortical aerenchyma enable enhanced root exploration by decreasing root metabolic costs that come with maintaining root biomass [58]. In potato, longer and more abundant stolon roots were associated with drought tolerance when genotypes were evaluated in the field [65].

**Figure 1:**
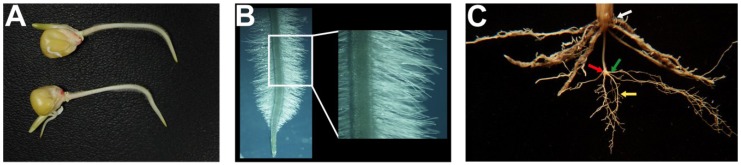
Root architectural, structural and anatomical traits for breeding crops with improved water and nutrient acquisition. (**A**) Root gravitropism in maize primary roots; (**B**) Extensive root hair formation on a wheat seedling; (**C**) Origin of root branches in wheat. New roots emerge from the leaf and coleoptile nodes (white and red arrows), seed (green arrow) and from primary/seminal roots (yellow arrow).

Another factor that specifies rooting depth and has relevance to crop breeding is gravitropism. Gravitropism is a physiological response manifested as a redirection of plant organ growth toward or away from the gravity vector (Figure 1A). The gravitropic response of roots was described in a recent study showing that the *DEEPER ROOTING 1* (*DRO1*) quantitative trait loci, which results in steeper root angles and more robust seedling gravitropic responses, leads to rice plants that are more tolerant to drought. The enhanced drought tolerance of *DRO1* plants is due to their deeper root system. The *DRO1* gene encodes a plasma membrane-localized protein that appears to be regulated by the plant hormone auxin, although the exact molecular function of DRO1 is still unknown [8]. Decades of research have led to the identification of several molecular components underlying root gravitropism including components of the auxin transport machinery, cell walls and the cytoskeleton [66,67,68]. One can envision strategies in which any of these components may be targets for improving rooting depth in crop plants.

Selection for faster growing and deeper roots could enhance the plant’s access to water deeper in the subsoil layers, enabling plants to maintain yield under limited rainfall conditions. Scenarios of water availability deep in the subsoil *vs.* periodic rainfall periods have different effects on crop growth and development, and these are reflected in the aboveground plant tissues as well as in the roots [69]. Despite the variations in the environment, in management practices and in the interactions between these two, some general root traits associated with crop productivity under drought have been described [34]. The size of a plant’s root system is a key trait that can affect the uptake of resources from the soil and should be considered in relation to the size of the aboveground plant parts (leaves, shoots and whole plant). The importance of a deep and vigorous root system for higher yield has been described for multiple species and previously summarized [31]. In some instances, however, recurrent selection for increased grain yield of maize under drought stress was associated with a decrease in root mass [70], suggesting the existence of a trade-off between allocating resources aboveground *vs.* belowground possibly due to a balancing metabolic cost for resource allocation. Shifts in the relationship between root and shoot can compensate for water limitations and impact the water status of plants. The metabolic costs would also differ between annual *vs.* perennial plant systems differing in length of lifespan.

### 2.2. Root Hairs

Root hairs are single cell projections that emerge from root epidermal cells (Figure 1B). Because root hairs account for a large percentage of the total root surface area, it comes as no surprise that they contribute to almost 50% of water absorption by the plant. The importance of root hairs for water uptake was shown by comparing wild-type Arabidopsis plants with a root hairless mutant. Due to the reduced ability of the root hairless mutants to absorb water, they are more sensitive to drought, salinity and heat stress [43]. In barley, root hairs have been shown to improve root penetration into hard, compact soils, a crucial trait for plant establishment as well as P acquisition [40]. Furthermore, a recent study in common bean (*Phaseolus vulgaris* L.) showed that the benefits of root hairs for crop productivity can be enhanced in cultivars with shallow basal roots. The combination of long root hairs and shallow basal roots results in a synergistic effect on P acquisition that translates to a 300% increase in biomass in cultivars with both traits [41]. In wheat, the formation of rhizosheaths (*i.e.*, soil that adheres to plant roots) is correlated with root hair tolerance to aluminum [44]. Several components of the molecular machinery that controls root hair growth are well known [71], and modifying the expression of some of the genes that control root hair development has led to plants with longer and highly branched root hairs [72,73]. However, whether such changes in root hair morphology result in more efficient water and nutrient uptake of these plants is yet to be determined (Table 1).

### 2.3. Root Branching

The formation of root branches or lateral roots is a significant determinant of overall RSA (Figure 1C). Lateral roots add to the total root biomass, total root length and root surface area. As such, it has been assumed that increased lateral root density is associated with greater nutrient and water uptake [5,74,75]. However, like many other root traits, the ideal lateral root density is dictated by the nutrient and water availability of the soil. Recent studies are evaluating the relationship between root branching and soil resource acquisition in greater depth. For instance, in soils where N is limiting, maize lines with long and few lateral roots have 30% more yield than those with many short lateral roots. Similar to the root anatomical traits discussed above, root systems with fewer lateral roots reduce the metabolic costs that come with having to maintain an elaborate root architecture and allocate more resources towards deeper roots to access N [48]. Alternatively, genotypes with more lateral roots are more adapted to low P soils [47] (Table 1), a conclusion also derived from transgenic studies where overexpression of β-expansin gene *GmEXPB2* in soybean [76,77], or wheat expansin gene *TaEXPB23* in tobacco [78], improved RSA and P efficiency as indicated by an increased number of lateral roots. The importance of total root length and root surface area for P acquisition and performance in low P soil is further evidenced through natural and transgenic alleles of *PHOSPHORUS STARVATION TOLERANCE 1* (*PSTOL1*) in rice and sorghum [7,79].

Basic studies on lateral roots have also uncovered mechanisms by which RSA is specified by the local soil environment. Many crops like wheat or maize develop different types of roots that include cluster, brace and crown roots that differ form the primary root in the node from which they grow [35,55,56,80]. Plants with these root systems can grow steep primary roots and shallow adventitious roots for a more efficient exploration of their surrondings, depending on the environmental conditions (Table 1). Roots of several plant species exhibit a phenomenon called hydropatterning, in which lateral roots emerge from the side of the root in contact with the moist soil [4]. Hydropatterning is not limited to lateral root formation since it is also observed in the emerging root hairs and aerenchyma formation. Additional studies are needed to better understand the adaptive value of hydropatterning and whether this newly discovered phenomenon can be exploited to improve water use efficiency and ultimately plant yield under drought [5].

## 3. Overview of Technologies Available for Phenotyping RSA Traits

Phenotyping root traits in the field is difficult, limiting the evaluation of RSA features and their use for selection during breeding [81]. Field-based techniques are also laborious and require plot destruction for sample collection. The heterogeneity in the soil structure and composition that can impact the RSA of field-grown plants at different sites within a field is another factor confounding the effect due to the genetic and the environment interactions. Alternative methods to root phenotyping in the field involve measuring roots in plants grown under a range of controlled conditions. The methods used to evaluate the architecture of plant roots should provide an accurate representation of the root growth, reduce the confounding effect of other environmental factors that could alter root growth, be of adequate throughput to phenotype a large number of genotypes routinely screened in plant breeding programs and enable the translation of root phenotypes from controlled environments to those of biological relevance when plants are grown in the field.

Several software packages have been developed for imaging roots and extracting quantitative data from captured root images. A few examples of these software tools include RootScan [82], RootNav [83], DART [84], GiARoots [85], IJ Rhizo [86], RootSystemAnalyzer [87], RootReader [88], RootReader3D [89] and RooTrak [90]. The growing number of image analysis tools dedicated to roots led to the recent development of Root System Markup Language (RSML) format to facilitate sharing of root architectural data between the different software packages and provide a standard format upon which to base centralized repositories of root trait data [91]. A detailed discussion on the various software packages for root image analysis is beyond the scope of this article and therefore we refer readers to an online resource (www.plant-image-analysis.org) and a recent review that surveys some of these tools [92].

Although downstream analysis of root images will be a crucial component of root phenotyping strategies, the method for culturing the plants often dictates the usefulness of any image analysis tools. The choice of plant cultivation system will depend on whether quantitative data on the root system will be used to answer basic root developmental questions or if the desired outcome is for high throughput root trait selection for breeding. For the former goal, the plant culture methods typically involve the use of artificial gel-based media or soil-filled containers and rhizotrons [28,93]. RSA features of a genotype can be analyzed under a range of controlled conditions to decipher genetic and environmental interactions. If the goal is for high throughput direct root selection and evaluation, plant cultivation methods range from screening young seedlings grown on germination paper to direct excavation of field-grown plants [94,95]. Each method has its advantages and disadvantages (Table 2). For example, gel-based systems, which allow direct visual access to the roots for imaging non-destructively in real time may present challenges in regard to their physiological relevance. These systems are suitable, however, for analyzing a large number of individuals or genotypes under a range of highly controlled conditions to ensure the repeatability of the experiments. Nondestructive imaging of roots in soil-filled containers would provide more physiologically relevant data and still allow testing the effects of specific environmental conditions. However, the soil-based methods are limited in throughput and the spatial/temporal resolution [90]. In addition, for laboratory and greenhouse-based methods, plant growth is evaluated without intra- or interspecific competitions, which may be relevant for studying root traits in the field. Examples of these methods include: Growth and Luminescence Observatory for Roots (GLO-Roots), X-Ray Computed Tomography and the clear pots method in greenhouse (Table 3). On the other hand, methods with high practical and physiological relevance use destructive assays. To a certain extent, shovelomics, soil coring methods and rhizolysimeters can minimize the loss of root structures (Table 3). All field-based methods are labor intensive and are subject to the effects of environmental variabilities in the field.

In summary, the method chosen for culturing plants for root imaging will depend on a suite of factors including the specific root trait of interest (e.g., primary roots *vs.* crown roots), the desired timescale for sampling (hours *vs.* days/months), infrastructure capacity and costs. Representative methods for growing plants for subsequent root imaging and phenotyping are presented in Table 3.

**Table 2:** Strategies and approaches for growing plants prior to root phenotyping.

Approach	Growth Conditions	Advantages	Disadvantages
Laboratory methods	Highly controlled	- Evaluate root growth in real time - Non-destructive - A large number of controlled growth conditions can be tested - Repeatable conditions - Large space for plant growth is not required - Easy to handle and clean roots	- RSA may be affected by the growth container - Sterile conditions for evaluation excludes effect of possible interaction with beneficial microbes - Plants are not exposed to environmental conditions and therefore physiological relevance of root phenes should be further evaluated
Greenhouse methods	Moderately controlled	- Intermediate system between lab and field - Enables control of certain conditions such as soil type and moisture, light intensity, temperature, pot sizes and water and nutrient inputs - Evaluate genetic potential of plant RSA without intraspecific competition	- Labor intensive to process and clean bigger roots - Plants could be exposed to some disease/insect pressure - RSA continues to be affected by the growth container - Plant performance evaluated in the absence of other plants and/or microorganisms in the soil unless experimental design includes it
Field methods	Minimally controlled	- Physiological and practical relevance	- Labor and time intensive - Challenges due to variability in the field, particularly for soil conditions - Intensive root clean-up - Destructive assays - Permits are required for evaluation of transgenic plants

**Table 3:** Strategies and approaches for root phenotyping.

Plant Cultivation System	Growth Media	Description	References
1. Growth and luminescence observatory for roots (GLO-Roots)	Soil (lab)	This method combines custom-made growth vessels and new image analysis algorithms to non-destructively monitor RSA development over space (2-D) and time. The technique allows information on soil properties (e.g., moisture) to be integrated with root growth data. The system makes use of luminescence imaging of roots expressing plant codon-optimize luciferase.	[96]
2. X-Ray computed tomography	Soil (lab and greenhouse)	Non-destructively visualizes opaque root structures by measuring the attenuation of ionizing radiation as it passes through the root. A series of projections are acquired and combined to reconstruct a 3D image of the root system.	[97,98]
3. Rhizophonics	Liquid media (lab)	Combines hydroponics and rhizotrons. System is made of a nylon fabric supported by an aluminum frame. The set-up is immersed in a tank filled with liquid media. Allows non-destructive, 2-D imaging of root architecture while simultaneously sampling shoots.	[99]
4. Clear pot method	Soil (greenhouse)	Uses transparent pots filled with soil or other potting media. Seeds are planted close to the pot wall to enable high- throughput imaging of roots along the clear pot wall. To prevent light exposure, the clear pot is placed in black pots while roots are developing.	[100]
5. Rhizoslides	Paper-based (lab, greenhouse)	The set-up consists of a plexiglass sheet covered with moistened germination paper. Seeds are planted on the slit of the plexiglass. The system allows separation of crown roots from embryonic roots.	[101]
6. Shovelomics	Soil (field-based)	Involves manual excavation of plants and separating roots from the shoots. Washed roots are then placed on a phenotyping board for root trait quantification. New algorithms allow extraction of several root traits in a high throughput manner.	[94,95]
7. Soil coring	Soil (field-based)	Uses a tractor-mounted, hydraulic soil corer to drive steel alloy sampling tubes into the soil. When combined with novel planting configurations (e.g., hill plots), this method allows for phenotyping deep rooted crop varieties.	[102]
8. Rhizolysimeters	Soil (field-based)	Elaborate facility consisting of an underground corridor and concrete silos and pipes to house soil-containing soil cores for direct root observation.	[103]
9. Minirhizotrons	Soil (field-based)	A transparent observation tube permanently inserted in the soil. Images of roots growing along the minirhizotron wall at particular locations in the soil profile can be captured over time.	[104,105]

## 4. Strategies for Root Phenotyping and Their Utilization in Breeding Programs

**Figure 2:**
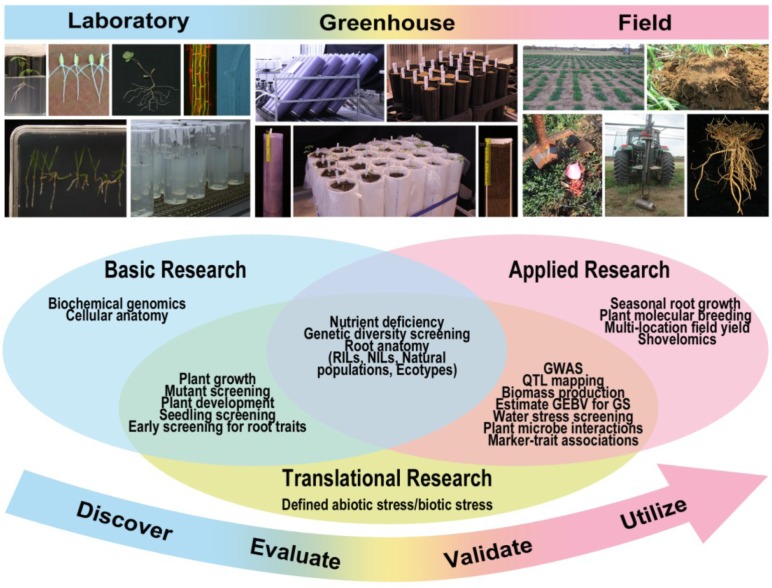
Overview of root phenotyping strategies in the greenhouse, laboratory and field and their application for basic, translational and applied research. The description of images is clockwise within each of the three categories. Laboratory: alfalfa (*Medicago sativa* L.) roots in clear vials and growth media; wheat (*Triticum aestivum* L.) seedlings growing on germination paper in plastic trays; alfalfa seedling imaged using flatbed scanner; *Arabidopsis thaliana* roots stained with propidium iodide to observe cell wall and green fluorescent protein (GFP) labeling the actin cytoskeleton; *A. thaliana* root clarified showing lateral root initiation; alfalfa seedlings growing in glass cylinders with growth media; *Brachypodium distachtyon* (model grass) seedlings growing in growth media in plates. Greenhouse: EnviroKing^®^ (Harrington Industrial Plastics, Albuquerque, NM, USA) UV clear PVC piping at a slanted angle; black deepots; semi-cylindrical mesocosm fronted with clear plexiglass; mesocosms with plastic liners; individual EnviroKing^®^ UV clear PVC piping for real-time observation of RSA including root depth. Field: overview of an alfalfa field trial; shovelomics approaches digging wheat roots; washed roots from field-grown alfalfa plants; tractor for obtaining core samples; outline of root area for harvest.

Developing plants with the capacity to grow and remain productive in marginal soils with reduced water and fertilizer inputs is a major target of crop and forage breeding programs worldwide. Although the identification of root traits and phenes that facilitate the exploration and effective utilization of water and nutrients can be used to achieve these breeding objectives, the challenge of phenotyping underground traits with higher throughput has hindered progress in this area. Regardless, crop breeding programs have increased yields by selecting for a combination of traits such as increasing shoot biomass, shifting the ratio between harvested grain *vs.* shoot biomass, improving disease resistance and expanding the length of the growing season. Yield increases through breeding have been associated with earlier flowering and decreased number of days between germination and harvest that could have resulted from inadvertently selecting for more efficient root systems. Understanding the variability and contribution of specific root traits, or phenes, in a given species will enable the identification of those traits capable of enhancing the efficiency of the root system more effectively, and that will result in increased plant productivity [106]. Success in targeted efforts to improve nutrient and water acquisition of crops by identifying and selecting for root ideotypes that are most suitable for a target set of soil and environmental conditions can be obtained using a combination of root phenotyping strategies that encompass laboratory, greenhouse and field evaluations (Figure 2). Prior knowledge of the RSA of different genotypes or breeding lines can be used to compare the productivity of a particular genotype in relation to root size when the plant is exposed to both nutrient and water deficits. The desirable root phenes may vary in different crops (annuals *vs.* perennials) and the integration of traits should consider possible tradeoffs from allocating resources to different plant organs (*i.e.*, root to shoot ratio). The heritability of the root phenes, the genotype-environment-management interactions and the temporal variations of various root types due to changing conditions (rainfall) also impact breeding decisions. Additionally, root phenotyping capabilities can enhance our understanding of the variation in the root systems of multiple species in response to various stress factors, aid in our assessment of how these systems impact the soil ecosystem and through this knowledge enable the development of strategies to modify RSA and improve soil health.

Reproducible and accurate phenotyping is also critical to identify quantitative differences in RSA of plant materials and identify the underlying genetic mechanisms (quantitative trait loci (QTL) and genes associated with root phenes) to implement genomics-assisted breeding strategies. The use of molecular breeding strategies depends on the phenotypic data used to determine the estimated breeding value of a particular allele in a given genetic background and set of environments. Several traits influencing root depth appear to be under the control of multiple genes [107,108], suggesting the potential for improvement/modification through selection and breeding. The presence of natural variation in root morphological traits in a target crop species also indicates that selection for specific root traits could be achieved. A number of genes involved in RSA are known either from gene mutants with quantifiable changes in primary root length, root branching, root hair formation or from QTL studies. However, the mechanistic details of how these QTL affect the root phenotype, the effect of the QTL in a different genetic background and their role in different soil types and environments, are comparatively limited.

Generally root phenotyping strategies in the laboratory are most useful for basic research activities as part of the discovery phase to identify genetic variation for RSA and understanding the genetics of root anatomy (Figure 2). The evaluation phase includes understanding the RSA and how the root anatomy and physiology changes in response to different abiotic and biotic stress in controlled single-variable experiments. Validation experiments are useful to determine the performance of plants with particular root phenes that enable evaluation of biomass/yield production and identification of associations between traits and molecular markers. The utilization stage includes utilizing molecular markers for selecting the desirable individuals to use as parents for crossing and population development, to track desirable root phenes during the breeding pipeline and further understand the plasticity of roots in multiple soil types under varying cultivation and crop management practices.

The prevalence of taproot *vs.* branched roots, the rate of root growth and the direction of root growth relative to limiting resources are important root traits that could be combined in breeding lines for future release as new cultivars if they can outperform existing cultivars in practical agricultural settings. Progress from phenotype-based selection has been achieved, however, without additional knowledge of the mechanisms involved. In alfalfa, for example, populations selected for more fibrous or lateral roots had greater biomass yield than populations selected for no or few fibrous or lateral roots [109]. Genetic gains from two cycles of divergent selection in alfalfa for root traits were achieved [110]. Additionally, studies have shown that the root morphology also influences the persistence and productivity of this perennial crop species [111]. In rice, root length, diameter, dry weight and total absorbing surface area were positively correlated with grain yield [112]. Also in rice, the DEEPER ROOTING 1 (DRO1) QTL controlling root growth angle was introgressed through backcrossing in a shallow-rooting cultivar. The resulting plants had deeper roots and higher yield performance under drought conditions [8]. It is worth noting that different root traits are important in the different types of drought that may occur at different developmental stages [69]. A major QTL for P starvation tolerance identified in rice, Pup1, led to the identification of a Pup1-specific protein kinase gene, *PSTOL1* [79]. PSTOL1 acts as an enhancer of early root growth thus enabling plants to acquire more P and other nutrients, resulting in enhanced grain yields in P-deficient soils. Additional strategies for breeding crop plants with deep roots for sustainable water, nutrient and carbon sequestration have been described elsewhere [6]. Specific root phenes and phene interactions can be estimated empirically through simulation modeling and near-isogenic lines that enable evaluating the effect of specific phene(s) in a common phenotypic background [13].

Screening a large number of individuals for root traits in order to incorporate root phenes that enhance water and nutrient acquisition as well as disease resistance into new germplasm is a continuous process and different phenotyping systems may serve different yet complementary roles. We propose here that researchers and breeders consider a combination of phenotypic root screening approaches. First, early root development traits from seedlings grown in controlled environments may be used for initial selections and to explore genetic (and heritable) factors underlying root traits, to identify parental genotypes. Then, the performance of individuals comparing for root traits in the greenhouse is evaluated and parents are selected to produce the next generation of plants. Lastly, multi-location field trials of advanced breeding lines are established to assess their performance (yield). This approach can accelerate the development of new cultivars with improved root systems (deeper roots, redistribution of branched roots and with greater hydraulic conductivity). Ultimately, breeders pursue phenotypes (yield, drought or salt tolerance, resistance to pests or diseases) that are either observed or quantified and reflect the interaction between the plant’s genotype and the conditions in which it is grown (genotype-environment interaction). The proposed approach will also serve to establish the relationship between young root systems suitable for rapid root screening assays in the laboratory or greenhouse, and mature root system vigor, water and nutrient uptake and, ultimately, higher yields in rain-fed systems. Selection systems for mature root system traits in the field are challenging and time-consuming and, hence, only a few methods for measuring roots in this environment are available. Root traits of young plants selected in the controlled conditions offer many advantages for screening although they should translate into mature root system traits in the field to be effective (Table 2). Fortunately, solid relationships between controlled-environment root vigor and field root vigor [34] have been identified. In the perennial species alfalfa, the ranking of genotypes based on various root traits were the same at the end of the seeding year (22 weeks after planting) and at the end of the first production year (74 weeks after planting) [113], suggesting that earlier evaluations are predictive of future root performance.

## 5. Perspectives and Conclusions

Drought, low soil fertility and soil-borne diseases are among the most widespread challenges for agricultural productivity on a global scale. Breeding crop and forage plants for specific root phenes (deeper, branched and responsively plastic) that are capable of adapting to variable growing conditions could improve water and nutrient acquisition, result in sustainable plant yields and improve soil structure and soil health. The value of research in controlled environments will be exemplified when the value of specific traits measured under these conditions is demonstrated in the field. Therefore, translating laboratory research to the field through the development of novel genotypes that integrate promising root traits into breeding lines that are adapted to grow in the target set of environments is a key step in the process. Breeders will adopt new screening methods only when the value of a specific root trait identified using high throughput phenotyping approaches performs well under field conditions. Suites of traits that benefit the acquisition of several essential mineral elements have been described [114] and these could be used as breeding targets to develop cultivars with the desired root phenes. Integrating root raits that benefit the acquisition of multiple essential mineral elements for plant growth may result in cultivars with root ideotypes suitable for growth in multiple environments under a variety of soil conditions.

It is possible that the desirable root ideotype is not a specific root phene representing a static or defined root phenotype, but rather the trait of interest refers to the plasticity of the roots that are able to respond to and readily adapt to a myriad of growing conditions (deep roots to capture water from the subsoil combined with root branching to increase the uptake of nutrients (*i.e.*, P) closer to the soil surface using a “topsoil foraging” root architecture). Strategies to measure and capture root plasticity will require evaluation of RSA and plant performance in a range of stress conditions commonly found simultaneously in the field (low soil moisture, high temperatures and/or limiting nutrients). The ideal RSA may differ for different crops at different locations due to phenotype–environment interactions as well as the specific agricultural management practices implemented in the field (*i.e.*, frequency of harvest or animal grazing). Although RSA has important functional implications for the acquisition of soil water and nutrients, direct selection for root architectural traits in breeding programs has previously been limited by the absence of suitable phenotyping methods with physiological relevance. Recent studies in potato, however, have identified significant correlations between greenhouse and field measurements for root characteristics and yield suggesting this approach could be feasible for both identifying genetic variation for root phenes and using greenhouse-based phenotyping strategies to increase yields in the field as part of the plant improvement process [115]. The utilization of a combination of root phenotyping strategies proposed here can be used to incorporate “root breeding” strategies aimed at enhancing plant performance through more efficient utilization of water and nutrients that will contribute to the sustainability of agricultural systems worldwide.
